# Changes in genetic diversity and differentiation in Red‐cockaded woodpeckers (*Dryobates borealis*) over the past century

**DOI:** 10.1002/ece3.5135

**Published:** 2019-04-08

**Authors:** Mark P. Miller, Julia T. Vilstrup, Thomas D. Mullins, Will McDearman, Jeffrey R. Walters, Susan M. Haig

**Affiliations:** ^1^ U.S. Geological Survey, Forest and Rangeland Ecosystem Science Center Corvallis Oregon; ^2^ Department of Fisheries and Wildlife Oregon State University Corvallis Oregon; ^3^ Division of Restoration and Recovery U.S. Fish and Wildlife Service Jackson Mississippi; ^4^ Department of Biological Sciences Virginia Tech Blacksburg Virginia; ^5^ Present address: Publons Wellington New Zealand

**Keywords:** *Dryobates borealis*, endangered species, microsatellite, mitochondrial DNA sequences, Red‐cockaded woodpecker, temporal change

## Abstract

Red‐cockaded woodpeckers (RCW; *Dryobates borealis*) declined after human activities reduced their fire‐maintained pine ecosystem to <3% of its historical range in the southeastern United States and degraded remaining habitat. An estimated 1.6 million RCW cooperative breeding groups declined to about 3,500 groups with no more than 10,000 birds by 1978. Management has increased RCW population abundances since they were at their lowest in the 1990s. However, no range‐wide study has been undertaken since then to investigate the impacts of this massive bottleneck or infer the effects of conservation management and recent demographic recoveries. We used mitochondrial DNA sequences (mtDNA) and nine nuclear microsatellite loci to determine if range‐wide demographic declines resulted in changes to genetic structure and diversity in RCW by comparing samples collected before 1970 (mtDNA data only), between 1992 and 1995 (mtDNA and microsatellites), and between 2010 and 2014 (mtDNA and microsatellites). We show that genetic diversity has been lost as detected by a reduction in the number of mitochondrial haplotypes. This reduction was apparent in comparisons of pre‐1970 mtDNA data with data from the 1992–1995 and 2010–2014 time points, with no change between the latter two time points in mtDNA and microsatellite analyses. The mtDNA data also revealed increases in range‐wide genetic differentiation, with a genetically panmictic population present throughout the southeastern United States in the pre‐1970s data and subsequent development of genetic structure that has remained unchanged since the 1990s. Genetic structure was also uncovered with the microsatellite data, which like the mtDNA data showed little change between the 1992–1995 and 2010–2014 data sets. Temporal haplotype networks revealed a consistent, star‐like phylogeny, suggesting that despite the overall loss of haplotypes, no phylogenetically distinct mtDNA lineages were lost when the population declined. Our results may suggest that management during the last two decades has prevented additional losses of genetic diversity.

## 1 INTRODUCTION

Most population genetic studies provide a snapshot of the current status of the species under investigation. Studies that adopt a historical perspective mainly do so using phylogenetic or phylogeographic concepts that provide insights about long‐term historical changes that may have occurred over evolutionary time scales (Avise, 2000). Advancements in molecular techniques have increased the feasibility of extraction and amplification of DNA from low yield and low‐quality sources such as historical museum specimens (Leonard, 2008b). These techniques have important practical applications within conservation genetics as they permit direct assessments of changes that have occurred over time through comparisons between historical and contemporary samples (D'Elia, Haig, Mullins, & Miller, 2016; Draheim, Baird, & Haig, 2012). Temporal changes in genetic diversity are of interest as the genetic history of a species can assist managers in predicting responses to stochastic and anthropogenic demographic fluctuations. Likewise, monitoring contemporary genetic diversity is important for evaluating the effects of current management and determining where to focus future efforts (Schwartz, Luikart, & Waples, 2007).

Red‐cockaded woodpeckers (RCW; *Dryobates borealis*; Chesser et al., 2018, recently changed from *Picoides borealis*) were widespread and abundant throughout the open, fire‐maintained Longleaf pine (LLP, *Pinus palustris*) ecosystem and other pine habitats in the southeastern United States prior to European settlement three centuries ago (Allen, Krieger, Walters, & Collazo, 2006; Peet & Allard, 1993). The LLP ecosystem harbors some of the most species‐rich communities in temperate North America (Mitchell, Hiers, O'Brien, Jack, & Engstrom, 2006; Peet & Allard, 1993) and is dependent on frequent natural fires every 1–10 years (Drew, Kirkman, & Gholson, 1998). Less than 3% of the estimated 24 million hectares of historical LLP ecosystem now remain as it went from a nearly continuous distribution across the southeastern coastal plains and adjacent areas to a highly fragmented condition due to habitat loss from timber cutting, other land use changes, and degradation of remaining habitat resulting from fire suppression (Allen et al., 2006; Kirkman & Jack, 2018; Mitchell et al., 2006). Based on historical accounts and the extent of habitat loss, it is clear that RCW underwent a massive population bottleneck between 1870 and 1930, which coupled with further declines driven by fire suppression from 1960 to 1980 resulted in extirpation of the species in the most northern regions of the species' range in Missouri, Maryland, Tennessee, and Kentucky (Conner, Rudolph, & Walters, 2001).

Red‐cockaded woodpeckers are monogamous, territorial cooperative breeders where male (and less often female) offspring frequently stay and assist with incubation and feeding of nestlings and fledglings (Haig, Walters, & Plissner, 1994), and therefore also delay their own dispersal and breeding (Walters, 1991). Most female and some male juveniles disperse during their first year, and though RCW do not disperse very far, it is generally females that disperse farther (Daniels & Walters, 2000; Kesler & Walters, 2012; Walters, 1991). RCWs are unusual in that they excavate roost and nest cavities in old (i.e., >80–120 years old), living pine trees. The excavation process is complex and apparently involves fungi introduced to the excavation by the birds (Jusino, Lindner, Banik, Rose, & Walters, 2016; Jusino, Lindner, Banik, & Walters, 2015), and typically takes many years to complete (Harding & Walters, 2004). As a result, cavities and the availability of old pines suitable for cavities limit current populations. Also, RCW rarely form new breeding groups by excavating cavities for new territories, but rather most nonbreeding adult helper individuals wait to fill breeding vacancies on their already established territories that contain a set of completed cavities (termed *cavity tree clusters*; Walters, Copeyon, & Carter, 1992). This promotes population stability, but also constrains population size and rates of population growth (Walters, 1991). In response, techniques for constructing artificial cavities in living pine trees were developed (Allen, 1991; Copeyon, 1990) as a means to sustain existing territories with natural cavity limitations and to create new viable territories at recruitment clusters, a management technique that has been highly successful in increasing the numbers of breeding groups in a population (Conner et al., 2001; Walters, Robinson, Starnes, & Goodson, 1995). Additional management techniques include habitat restoration through prescribed burning and translocation of birds among populations. The first successful translocations of individual birds were conducted in 1986 at Savannah River Site (Franzreb, 1999; Haig, Belthoff, & Allen, 1993a). Since the mid‐1990s, birds have been translocated annually from select larger donor populations to augment size and growth of smaller, isolated recipient populations (Costa & DeLotelle, 2006).

Historical RCW population numbers have been estimated at more than 1.6 million cooperative breeding groups (Conner et al., 2001) decreasing to 3,500 groups and <10,000 total birds by 1978 (Jackson, 1978) shortly after they became one of the first species protected under the U.S. Endangered Species Act. RCW continued to decline during the 1970s and 1980s (James, 1995; USFWS, 2003). By the early 2000s, RCW populations had increased to an estimated 5,627 active territories and approximately 14,000 birds (USFWS, 2003), indicating population stabilizations and increases in some populations due to new management programs (Costa, 2004; Rudolph, Conner, & Walters, 2004). Today, there are at least 7,800 active territories (W. McDearman, personal communication) in response to successful recovery management.

We sampled range‐wide from available RCW museum specimens, previously collected blood samples, and contemporary RCW populations to determine the extent that genetic diversity and structure have changed in RCW populations over the past century. The first and most recent range‐wide population genetic studies of RCWs were conducted in the early 1990s when population abundances were at their lowest (Haig, Belthoff, & Allen, 1993b; Haig, Bowman, & Mullins, 1996; Haig, Rhymer, & Heckel, 1994; Stangel, Lennartz, & Smith, 1992). These studies applied allozyme, DNA fingerprints, and randomly amplified polymorphic DNA (RAPD) markers and found low genetic diversity, especially in smaller populations. However, intensive management efforts have been applied to this species for over two decades since then (Baker, 1999; Ferraro, McIntosh, & Ospina, 2007; Leonard 2008a), and mitochondrial and microsatellite markers developed for RCWs are now available (Alstad 2010; Fike, Athrey, Bowman, Leberg, & Rhodes, 2009). Thus, an updated assessment of genetic structure and diversity in RCWs may illustrate if genetic patterns have changed over time and provide insight into whether management actions have had an impact on population genetic parameters.

## 2 METHODS

### 2.1 Sample collections

**Figure 1:**
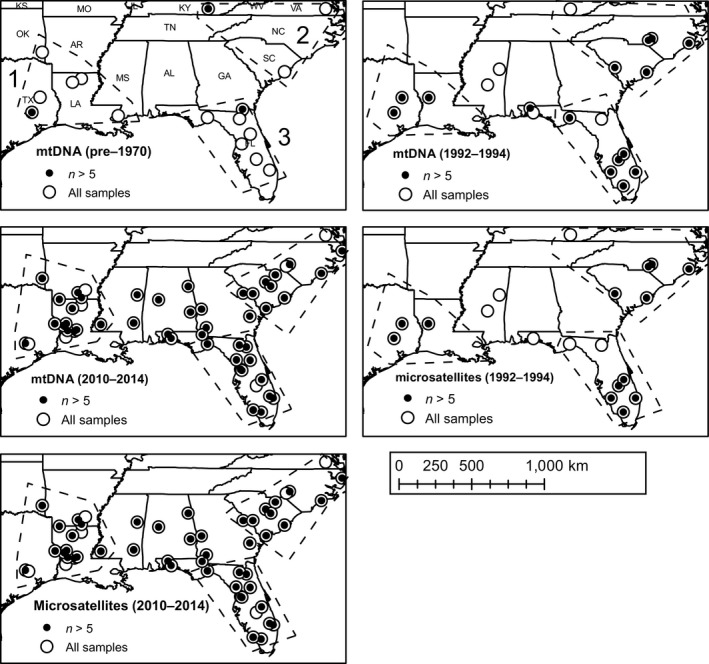
Maps highlighting the spatial distribution of Red‐cockaded Woodpecker sampling locations throughout the southeastern United States. Individual maps illustrate differences in sample sets for mtDNA versus microsatellite data sets as well as differences among pre‐1970s data, data from 1992–1995, and data from 2010–2014. Open circles highlight areas where fewer than five individual samples were available, whereas filled circles show locations where data for five or more samples existed. Dotted outlines show the boundaries for three regional sampling groupings defined by locations of samples from the pre‐1970s data or for comparable groupings of available sample locations from later years (see Supporting Information Appendix S1 for more information). Region 1: Western; Region 2: Eastern; Region 3: Florida.

We assembled a collection of RCW blood and tissue samples to use as a source of DNA for this study (Figure 1). The samples encompassed three distinct time periods (Supporting Information Appendix S1), thereby permitting us to take a temporal perspective and identify changes in genetic structure and diversity over the past century. First, we acquired toepad tissue samples from 50 historical museum specimens located in 13 university and natural history museums across the United States. Samples represented by this group were originally field collected before 1970 (range: 1881–1969; Supporting Information Appendix S2) with 95% of samples collected prior to 1950 (Supporting Information Appendix S3). Second, we analyzed a collection of 123 blood samples previously obtained from RCW between 1992 and 1995 during a point in time close to the onset of intensive management of RCW populations and restoration of longleaf and other open pine habitat in the southeastern United States. Finally, 513 blood and buccal swab samples were collected between 2010 and 2014, with buccal swab samples obtained using the protocol outlined in Vilstrup et al. (2018). These latter samples permitted us to characterize contemporary patterns across the RCW range. All sampling was performed under permits provided by the U.S. Fish and Wildlife Service to individual scientists and biologists.

### 2.2 DNA extraction

DNA extraction protocols varied depending on sample type (museum vs. blood vs. buccal swab samples). DNA was obtained from buccal swabs using the protocol outlined in Vilstrup et al. (2018). Blood samples were extracted using the DNeasy Blood and Tissue kit (Qiagen, Inc.) with 10–20 µl blood as input and elution in 100 µl AE buffer preheated to 37°C prior to the elution spin. Museum samples were initially soaked in ddH_2_0 for 24 hr to remove potential inhibitors and then extracted using the same general protocol used for the buccal swabs, with the exception that tissue digestion occurred in a three‐step process that included: (a) a preliminary overnight incubation with proteinase K as per the buccal swab protocol, (b) a manual grinding step using plastic mortars, and (c) an additional overnight incubation after addition of 10 µl Proteinase K. DNA extractions for the museum samples were performed in a separate laboratory from the blood and buccal swab samples.

### 2.3 Mitochondrial DNA and microsatellite data

Three mitochondrial markers (Cytochrome b, Cytochrome oxidase subunit I, and the control region) were initially screened for a subset of samples spanning the species' range. We found very low genetic diversity at the Cytochrome b and COX1 marker, and therefore, chose to only sequence part of the hypervariable region of the control region (CR) for the remaining samples. Primers were designed to amplify approximately 800 base pairs (bp) of the CR based on prior available sequences (Alstad 2010; Supporting Information Appendix S4). Five overlapping primer pairs amplifying 100–240 bp each were needed to amplify the museum samples and were, in a few cases, also used to sequence some of the blood and buccal swab samples (Supporting Information Appendix S4). Polymerase Chain Reactions (PCR) were performed with a total volume of 25 µl that consisted of 1–3 µl diluted DNA extract, 1X PCR buffer, 2 mM MgCl2, 10 mM dNTPs, 0.4 mM of each primer, and 0.035 U of Taq Gold. The cycling conditions included a 5‐min initial denaturation at 95°C, followed by 35–40 cycles of 30 s denaturation at 95°C, 30 s annealing at 51–55°C depending on primer, and 50 s elongation at 72°C, with a final elongation at 72°C for 10 min. Blank controls were included with every PCR to monitor for contamination. Primer extension and bidirectional sequencing of the PCR products was performed using the amplification primers and ABI Big Dye sequencing chemistry on an ABI 3700 automated DNA sequencer. Sequences from each individual were assembled and aligned in Geneious v.8.0.2 (Kearse et al., 2012) and visually inspected before trimming to a final sequence length of 605 bp. Final mtDNA data sets contained 50 individuals from 16 locations for the historical data set, 123 individuals from 20 locations for the 1992–1995 data set, and 513 individuals from 53 locations for the 2010–2014 data set (Figure 1 and Supporting Information Appendix S1).

Nine microsatellite markers developed for RCW (Fike et al., 2009; primers RCW01, RCW03, RCW06, RCW12, RCW20, RCW22, RCW28, RCW40, and RCW42) were genotyped for the blood and buccal swab samples that encompass the 1992–1995 and 2010–2014 time periods. Historical samples derived from museum toe pads did not amplify reliably. PCR primers were labeled with 5' 6‐FAM or HEX. PCR reactions were performed in 10 µl volumes consisting of 2–3 µl diluted DNA extract, 1X PCR buffer, 2 mM MgCl2, 10 mM dNTPs, 0.5 mM each of regular forward and reverse primer, 0.05 mM dye‐labeled forward primer, and 0.05 U Go Taq Flexi. The cycling conditions were 2 min initial denaturation at 94°C, followed by 32 cycles of 30 s denaturation at 94°C, 30 s annealing at 64/65°C, and 30 s elongation at 72°C, with a final elongation at 72°C for 10 min. Genescan was performed using an ABI 3730 capillary DNA automated sequencer with ROX 400 size standard. All PCRs included blank controls to monitor contamination, and successful amplification was determined by running 3 µl amplified extract on a 1% agarose gel. Microsatellite peaks were scored in Geneious v.8.0.2 (Kearse et al., 2012). The presence of null alleles, stuttering, or large allele drop out was evaluated for each locus using the program Micro‐Checker (Oosterhout, Hutchinson, Wills, & Shipley, 2004). Samples where more than four loci failed to amplify were excluded from analysis, though we obtained genotypes at all loci for 81% of the samples that were included in the final data set. The final microsatellite data included 118 individuals from 19 different locations in the 1992–1995 data set, and 504 individuals from 53 locations for the 2010 to 2014 data set (Figure 1, Supporting Information Table S1).

### 2.4 Changes in genetic diversity over time

Data sets included in this study contained varying sample sizes and differences in the locations from which samples originated (Figure 1 and Supporting Information Appendix S1). Thus, given our goal of identifying changes in genetic diversity over time, we performed a number of analyses using various partitions, groupings, and subsets of the data to help equalize data sets and make them as comparable as possible among different time points (see group definitions below). For the mtDNA data, we used Arlequin version 3.5 (Excoffier & Lischer, 2010) to quantify the observed number of haplotypes in each group (*A*_mtDNA_), haplotype diversity (*H*), and nucleotide diversity (*π*). Although haplotype diversity and nucleotide diversity are unbiased estimators whose values are not affected by sample size, the observed number of haplotypes is potentially correlated with sample size (Kalinowski, 2004). Given that the pre‐1970 and 1992–1995 data were derived from smaller numbers of samples relative to the 2010–2014 data, we used the program HP‐Rare (Kalinowski, 2005) to obtain rarefied estimates of the number of haplotypes for each group (*Ar*_mtDNA_) by assuming sample sizes associated with the smallest group of interest in a particular comparison (Tables 1, 2, 3, 4).

**Table 1:** Mitochondrial DNA (mtDNA) genetic diversity in Red‐cockaded Woodpeckers across data sets and within three regions (Figure 1; 1: Western, 2: Eastern, 3: Florida) at three different time points

	Genetic diversity				
	*n*	*π*	*H*	*A*	*Ar*_mtDNA_
All data					
Pre−1970	50	0.0022	0.766	22	22.00
1992–1995	123	0.0027	0.789	26	15.17
2010–2014	501	0.0020	0.716	42	16.30
Region 1 (Western)					
Pre−1970	17	0.0021	0.662	7	7.00
1992–1995	21	0.0005	0.186	3	2.62
2010–2014	151	0.0020	0.655	16	6.52
Region 2 (Eastern)					
Pre−1970	12	0.0021	0.833	7	7.00
1992–1995	50	0.0019	0.711	11	4.71
2010–2014	94	0.0018	0.677	11	4.44
Region 3 (Florida)					
Pre−1970	21	0.0025	0.824	12	12.00
1992–1995	40	0.0043	0.869	12	8.81
2010–2014	135	0.0024	0.809	18	8.98

Note

*A*: haplotype richness; *Ar*_mtDNA_: rarefied estimate of haplotype richness accounting for differences in sample sizes at different time points; *H*: haplotype diversity; *n*: sample size; *π*: nucleotide diversity.

**Table 2:** Mitochondrial DNA (mtDNA) genetic diversity in Red‐cockaded Woodpeckers within ecoregions (Supporting Information Appendix S5)

Ecoregion	Date range	Genetic diversity				
		*n*	*π*	*H*	*A*	*Ar*_mtDNA_
CUMB	Pre−1970	8	0.0015	0.750	4	—
EGCP	1992–1995	12	0.0037	0.970	10	10.00
	2010–2014	96	0.0014	0.645	13	5.00
MACP	1992–1995	16	0.0019	0.725	5	5.00
	2010–2014	38	0.0016	0.653	7	4.89
SACP	Pre−1970	10	0.0036	0.978	9	9.00
	1992–1995	13	0.0016	0.654	4	3.54
	2010–2014	41	0.0014	0.612	8	4.06
SAND	1992–1995	20	0.0022	0.590	8	8.00
	2010–2014	62	0.0023	0.737	11	6.73
SCF	Pre−1970	10	0.0016	0.667	5	5.00
	1992–1995	32	0.0047	0.849	8	5.49
	2010–2014	93	0.0027	0.836	14	5.85
UEGCP	1992–1995	6	0.0017	0.800	4	4.00
	2010–2014	30	0.0017	0.628	8	3.11
UWGCP	Pre−1970	12	0.0021	0.758	6	3.34
	1992–1995	5	0.0000	0.000	1	1.00
	2010–2014	38	0.0017	0.401	5	1.99
WGCP	1992–1995	16	0.0006	0.242	3	3.00
	2010–2014	103	0.0021	0.722	13	6.51

Notes

*A*: haplotype richness;* Ar*_mtDNA_: rarefied estimate of haplotype richness accounting for differences in sample sizes at different time points; *H*: haplotype diversity;* n*: sample size; *π*: nucleotide diversity.

Diversity statistics are reported only for time points and ecoregions where sample sizes of 5 or more were available. Ecoregion abbreviations are as follows: CUMB = Cumberlands; EGCP = East Gulf Coastal Plain; MACP = Mid Atlantic Coastal Plain; SACP = South Atlantic Coastal Plain; SAND = Sandhills; SCF = South Central Florida; UEGCP = Upper East Gulf Coastal Plain; UWGCP = Upper West Gulf Coastal Plain; WGCP = West Gulf Coastal Plain.

**Table 3:** Microsatellite genetic diversity in Red‐cockaded Woodpeckers across data sets and within three regions (Figure 1; 1: Western, 2: Eastern, 3: Florida) at three different time points

Data partition	Date range	Genetic diversity				
		*n*	Ho	He	*A*	*Ar*_microsat_
All data	1992–1995	115	0.406	0.436	3.11	3.11
	2010–2014	499	0.393	0.436	3.67	3.32
Region 1 (Western)	1992–1995	21	0.407	0.443	2.56	2.56
	2010–2014	151	0.410	0.453	3.44	2.77
Region 2 (Eastern)	1992–1995	49	0.415	0.435	2.89	2.89
	2010–2014	100	0.381	0.411	3.00	2.92
Region 3 (Florida)	1992–1995	38	0.395	0.428	2.78	2.78
	2010–2014	124	0.384	0.411	3.44	3.06

Note

*A*: average allelic richness over loci; Ar_microsat_: rarefied allelic richness accounting for sample size differences; He: expected heterozygosity; Ho: observed heterozygosity; *n*: sample size.

**Table 4:** Microsatellite genetic diversity in Red‐cockaded Woodpeckers within ecoregions (Supporting Information Appendix S5) during two time periods

Ecoregion	Date range	Genetic diversity				
		*n*	Ho	He	*A*	*Ar*_microsat_
EGCP	1992–1995	5	0.563	0.481	2.13	2.00
	2010–2014	99	0.372	0.430	3.22	2.33
MACP	1992–1995	16	0.368	0.423	2.78	2.78
	2010–2014	39	0.419	0.441	2.67	2.60
SACP	1992–1995	13	0.382	0.404	2.56	2.56
	2010–2014	42	0.395	0.416	2.89	2.59
SAND	1992–1995	19	0.462	0.438	2.56	2.56
	2010–2014	67	0.361	0.402	3.00	2.73
SCF	1992–1995	32	0.382	0.427	2.78	2.78
	2010–2014	81	0.372	0.409	3.33	3.06
UEGCP	1992–1995	6	0.407	0.416	2.56	2.56
	2010–2014	30	0.436	0.460	2.78	2.40
UWGCP	1992–1995	5	0.525	0.489	2.38	2.22
	2010–2014	39	0.403	0.431	2.78	2.24
WGCP	1992–1995	16	0.390	0.430	2.56	2.56
	2010–2014	102	0.423	0.450	3.22	2.68

Note

A: average allelic richness over loci; Ar_microsat_: rarefied allelic richness accounting for sample size differences; He: expected heterozygosity; Ho: observed heterozygosity; *n*: sample size.

See Table 2 for ecoregion abbreviations.

Genetic diversity was quantified using three approaches for aggregating samples into groups to promote the ability to make comparisons among time points. First, the genetic diversity parameters described above from each time period were analyzed *en mass* to provide an overall sense for whether genetic diversity has changed among the pre‐1970 data, the 1992–1994 data, and the 2010–2014 data. For the rarefied estimate of number of haplotypes, estimates for the 1992–1994 and 2010–2014 periods were obtained using assumed sample sizes of 50 (the total sample size of the pre‐1970 data set). Second, samples from the pre‐1970s mtDNA data set could be grouped into three distinct geographic regions based on locality information associated with the museum specimens (Western, Eastern, and Florida; Figure 1 and Supporting Information Appendix S1). Thus, we used comparable post hoc groupings of collection sites with similar spatial extents as the basis for making comparisons with approximately congruent geographic groupings of sample locations from the 1992–1995 and 2010–2014 data set (Figure 1). These comparisons allowed us to determine if there was spatial variation associated with any temporal genetic diversity changes detected in the analyses of the samples *en mass* as described above. As with the analyses of the complete data sets, rarefied estimates of the number of unique haplotypes for the 1992–1994 and 2010–2014 data were obtained using the regional sample size associated with each group in the pre‐1970 data set (Table 1). Finally, the range of RCW spans 10 distinct level III ecoregions that define a variety of biomes across the southeastern United States (Omernick, 1995; Supporting Information Appendix S1 and S5) and are important recovery units in the management of RCW populations (USFWS, 2003). Thus, an additional set of comparisons was made by pooling sampling locations from each time point at the ecoregion level to account for these different areas and the potential effects that they may have on the genetics of the RCW study system. In the case of the pre‐1970 data, aggregation at this fine scale resulted in very low sample sizes within many ecoregions. We therefore established a cutoff sample size of *n* = 5 to determine whether each ecoregion had sufficient data, leaving three to be included in our analyses (ecoregions South Atlantic Coastal Plain, South Central Florida, and Upper West Gulf Coastal Plain; Supporting Information Appendix S1 and S5). As with the other partitions of the data, the number of individuals from the time period with the smallest sample size in a given ecoregion (Table 2) was used when calculating rarefied estimates of the number of haplotypes. Note that because not all ecoregions were represented in the pre‐1970 data, comparisons at the ecoregion level were only possible in most cases between the 1992–1994 and 2010–2014 data sets.

For comparisons between the two microsatellite data sets (1992–1994 and 2010–2014 data sets only), we used Arlequin 3.5 (Excoffier & Lischer, 2010) to calculate observed and expected heterozygosity (*H*_o_ and *H*_e_, respectively) and the average number of alleles per locus (*A*_microsat_), recognizing that the latter quantity is also affected by sample sizes. Thus, rarefaction‐based analyses as described above were used to estimate *Ar*_microsat_ and correct the number of microsatellite alleles for the time period with the smaller sample size in our comparisons. These diversity statistics were calculated for the complete data sets, within each of 3 post hoc groupings defined by the pre‐1970 mtDNA data (Figure 1), and for each of eight ecoregions (Supporting Information Appendix S5) where sample sizes were >5. In all microsatellite analyses, individuals were included only if genotypes (nonmissing data) were available at 5 or more out of the 9 loci.

### 2.5 Changes in genetic structure over time

We compared the magnitude of genetic structure (*F*_ST_) at different time points using the Analysis of Molecular Variance (AMOVA; Excoffier, Smouse, & Quattro, 1992) procedure as implemented in Arlequin 3.5 (Excoffier & Lischer, 2010). Analyses of mtDNA included information on molecular differences between individual haplotypes as quantified by the proportion of nucleotides that differ between DNA sequences. Because of differences in sampling locations and sample sizes associated with the three mtDNA data sets (pre‐1970, 1992–1994, and 2010–2014) described above, differentiation was calculated for various comparable partitions of the data similar to our analyses of genetic diversity. For the mtDNA data, we quantified genetic differentiation for each data set among the three ad hoc regions (Figure 1), among collection areas where five or more samples were available (Figure 1), and among ecoregions where aggregated sample sets had sample sizes of 5 or more.

Microsatellite data sets (1992–1994 and 2010–2014) were analyzed using the locus‐by‐locus analysis option in Arlequin, resulting in a measure of differentiation comparable to Weir and Cockerham's (1984) unbiased estimator of *F*_ST _(Weir and Cockerham's θ). As with analyses of the mtDNA data, separate estimates of differentiation were quantified for the 3 ad hoc regions, ecoregions where aggregated samples had sample sizes of 5 or more, and sample locations where data from 5 or more individuals were available.

We used two additional approaches to test for changes in differentiation patterns over time. For mtDNA data, we used the TempNet package (Prost & Anderson, 2011) for R (R Core Team, 2013) to generate a temporal haplotype network and determine if phylogenetic lineages have been potentially lost over the time periods encompassed by the mtDNA data. Likewise, we also used the Bayesian clustering procedure implemented in the program STRUCTURE version 2.3.4 (Pritchard, Stephens, & Donnelly, 2000) to analyze the nuclear microsatellite data. This procedure identified putative genetic clusters of individuals and probabilistically assigned each individual to one of the identified clusters. The STRUCTURE analyses also allowed us to determine if changes in genetic structure patterns occurred between the 1992–1994 and 2010–2014 time periods. Analyses were performed separately for each data set using the admixture model and correlated allele frequency models, as recommended by Falush, Stephens, and Pritchard (2003), using values of *K* (the assumed number of clusters) ranging from 1 to 5. Five replicate analyses were performed for each value of *K* using an initial burn‐in of 10^5^ Markov‐Chain Monte Carlo steps followed by recording for 10^6^ steps. We calculated the average likelihood associated with runs from each value of *K* and assumed that values of *K* with the highest average likelihood score reflected the true number of genetic clusters.

## 3 RESULTS

We detected 66 unique mtDNA haplotypes across all individuals included in our data sets (Genbank Accession Numbers MK253579–MK253645). Our analyses suggested that mitochondrial haplotypes have been lost in RCWs over time. Across the complete data sets, no consistent changes in *π* or *H* were apparent, however, rarefied estimates of haplotype richness (*Ar*_mtDNA_) that accounted for different sample sizes from each time period were lower in the 1992–1995 and 2010–2014 data sets relative to the pre‐1970 data (Table 1). Analyses of the Western, Eastern, and Florida regions (Figure 1) revealed a similar pattern for *Ar*_mtDNA_ in two of the three regions examined (Eastern and Florida, Regions 2 and 3 in Figure 1; Table 1), whereas diversity statistics within the Western region (region 1) demonstrated a reduction in 1992–1995 relative to the pre‐1970 data and a return to pre‐1970 levels in 2010–2014. At the ecoregion level, evidence existed for reductions in *Ar*_mtDNA_ in the South Atlantic Coastal Plain and Upper West Gulf Coastal Plain (Table 2), whereas no apparent reduction occurred within South Central Florida. A potential decrease was also detected within the East Gulf Coastal Plain between the 1992–1995 and 2010 and 2014 data sets, whereas an increase during this period was identified in the West Gulf Coastal Plain (Table 2). The microsatellite data, which were restricted to information from 1992–1995 and 2010–2014, revealed no overt changes in genetic diversity patterns at any spatial scale during these time periods (Tables 3 and 4).

Based on the mtDNA data, quantitative measures of genetic differentiation were lower at all spatial scales for the pre‐1970s data relative to data sets representing 1992–1995 and 2010–2014 (Table 1). Nonsignificant F_ST_ values were calculated for the pre‐1970 data, and point estimates were slightly negative reflecting the absence of genetic structure. By contrast, and regardless of the sample groupings used for analysis, estimates of F_ST_ for the 1992–1995 and 2010–2014 mtDNA data were significantly larger than zero and relatively comparable between the two time periods (Table 5). The microsatellite data likewise illustrated the existence of genetic structure for the 1992–1995 and 2010–2014 data, with the magnitude of differentiation also relatively comparable between those two time periods (Table 5).

**Table 5:** Genetic differentiation patterns among Red‐cockaded Woodpeckers at different spatial scales including local sampling units, ecoregions (Supporting Information Appendix S1), and regional groupings (Figure 1)

Data source and grouping	Overall *F*_ST_
3 regional groups	
mtDNA (pre−1970)	−0.004
mtDNA (1992–1995)	0.087*
mtDNA (2010–2014)	0.061*
Microsatellites (1992–1995)	0.018*
Microsatellites (2010–2014)	0.018*
Available locations (*n* > 5)	
mtDNA (pre−1970)	−0.032
mtDNA (1992–1995)	0.171*
mtDNA (2010–2014)	0.208*
Microsatellites (1992–1995)	0.060*
Microsatellites (2010–2014)	0.053*
8 Ecoregions	
mtDNA (pre−1970)	−0.008
mtDNA (1992–1995)	0.071*
mtDNA (2010–2014)	0.062*
Microsatellites (1992–1995)	0.020*
Microsatellites (2010–2014)	0.018*

Notes

Results are presented for mtDNA data and data from analyses of nine nuclear microsatellites in data sets derived from pre‐1970s samples, samples collected in 1992–1995, and samples from 2010–2014.

* Statistical significance (*p* < 0.001).

**Figure 2:**
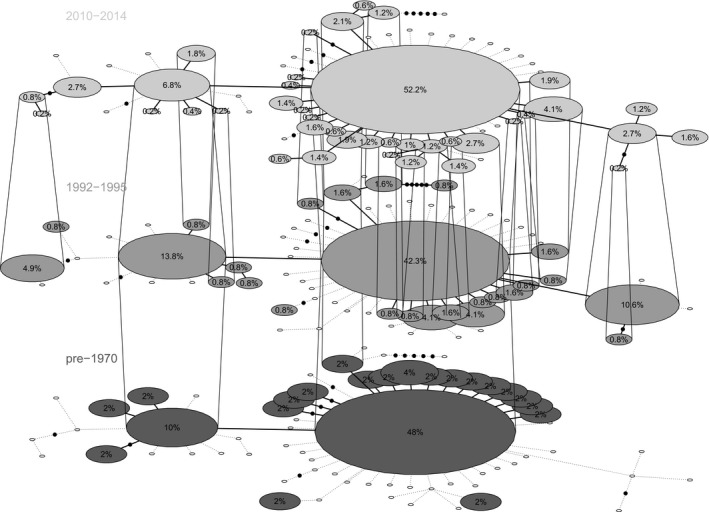
Temporal haplotype network showing shared haplotypes between the three sampled time periods for Red‐cockaded Woodpeckers. Numbers within circles represent the percent of individuals with haplotype. Clear circles represent the absence of a haplotype from the time layer and black dots represents one additional mutational step between haplotypes

Temporal haplotype networks generated for the mtDNA data (Figure 2) identified star‐like phylogenies at all three time points and revealed no loss of highly divergent phylogenetic lineages within the species. Analyses with STRUCTURE indicated that the *K* = 1 solutions were most likely for the 1992–1995 and 2010–2014 microsatellite data sets, which further highlighted the similarity of genetic structure patterns between time points and reiterated that deep genetic differentiation patterns did not exist (Supporting Information Appendix S6).

## 4 DISCUSSION

The availability of historical museum samples for use in retrospective comparisons with contemporary samples allows researchers to determine the extent of genetic changes that occur within a species over time. However, the inability to plan the specific sampling locations of historical samples poses its own set of challenges. Completely balanced data sets are unlikely, and it can be difficult to obtain historical data from all regions of interest (Draheim et al., 2012). However, in our study, we were able to use a number of different hierarchical spatial groupings of the samples that were collected at different time points (Figure 1 and Supporting Information Appendix S1) to help illustrate the degree that RCW genetic structure and diversity have changed over the last century.

Overall, our mtDNA data point to changes in genetic differentiation patterns and the existence of greater genetic diversity in RCW prior to the population declines that it experienced in the early to mid‐20th century (Table 1). This loss of diversity is consistent with many other investigations that have documented a loss of alleles in declining species of management interest (Wandeler, Hoeck, & Keller, 2007) and is not surprising given the magnitude of the population bottleneck experienced among RCW populations. Consistent with the results from Hoban et al. (2014), we primarily identified differences in the number of mitochondrial alleles versus other measures of genetic diversity (Tables 1 and 3). Hoban et al. used simulations to demonstrate that this metric (number of alleles, or number of haplotypes by extension) outperforms other diversity measures for documenting cases of genetic erosion.

The loss of diversity was only observed in our mtDNA data set because the microsatellite loci did not reliably amplify from the museum specimens that we examined (Tables 3 and 4), thereby precluding them from direct evaluation over the same time scales encompassed by the mtDNA. Other studies have illustrated genetic responses to population bottlenecks by microsatellite loci. Bouzat, Lewin, and Paige (1998) demonstrated a loss of microsatellite alleles in comparisons of contemporary and museum‐derived samples of Greater Prairie Chickens (*Tympanuchus cupido*) that were known to have been reduced to a population of ~50 individuals in 1993 (Bateson et al., 2014). Likewise, comparisons of historical and contemporary specimens of the Mauritius Kestrel (*Falco punctatus*) identified a similar loss of microsatellite alleles (Groombridge, Jones, Bruford, & Nichols, 2000). Sonsthagen, Wilson, and Underwood (2017) identified genetic effects of population bottlenecks at both mtDNA and microsatellite loci for Hawaiian Coots (*Fulica alai*) and Hawaiian Gallinules from Hawaii (*Gallinula galeata sandvicensis*). While these studies illustrate that similar patterns may be revealed by mtDNA and microsatellite markers, it does not guarantee that a comparable scenario exists in RCW. Mitochondrial DNA, by virtue of being haploid and maternally inherited, has a fourfold smaller effective population size relative to diploid, biparentally inherited nuclear loci. Consequently, mtDNA is more sensitive to the effects of genetic drift and may show more rapid or dramatic changes compared to loci such as microsatellites. At this time, given the absence of microsatellite data from the pre‐1970s data set, we are unable to conclusively state that nuclear genetic diversity has been reduced.

Although our analyses indicated that mtDNA haplotypes have been lost in RCW, our analysis was based on a fragment of the mitochondrial genome. Recent work compared the apparent loss of diversity in a known population bottleneck of Giant Galapagos tortoises (*Chelonoidis nigra*) between data sets based on mitochondrial gene fragments, sets of mitochondrial genes, or complete mitochondrial genomes (Jensen et al., 2018). One outcome of their investigation was the suggestion that a reliance on analyses of single genes could overestimate the magnitude of loss compared to analyses based on complete mitochondrial genomes. Use of targeted capture laboratory techniques to facilitate generation of complete mitochondrial genomes, as performed by Jensen et al. (2018), could enhance our ability to generate such information from archival samples and help clarify the generality of their findings in this and other study systems.

Comparisons of the 1992–1995 and 2010–2014 data derived from the mtDNA and microsatellites suggested that, with a few exceptions, similar genetic diversity levels existed at these two time points (Tables 1, 2, 3, 4). More effective management of RCW populations began in the early 1990s to alleviate cavity limitations with artificial cavities, induce new breeding groups at recruitment clusters provisioned with artificial cavities, sustain and increase habitat by compatible forest and prescribed fire management programs, and translocate individuals to bolster population sizes or re‐establish extirpated populations throughout its range (Conner et al., 2001; Costa & DeLotelle, 2006; Kulhavy et al., 1995). Given that we have observed minimal changes in diversity parameters between the 1992–1995 and 2010–2014 time periods, it remains feasible that these management actions helped halt the loss of diversity detected in comparisons with the earlier time period (Tables 1, 2). Indeed, in our analyses, the Western Region was characterized by a decline in the 1992–1995 mtDNA data followed by an apparent recovery to historical haplotype abundance levels in 2010–2014. We note that Region 1 includes the West Gulf Coastal Plain (WGCP) ecoregion (Supporting Information Figure S1), which also demonstrated an increase in mtDNA haplotypes between 1992–1995 and 2010–2014 when examined separately (Table 2). This result may point to an even more substantial effect of management actions on RCW populations in some portions of their range. For example, 11 of the 17 sampling locations for the 2010–2014 period in the Western Region have been translocation recipients (W. McDearman personal communication). Translocations have long been used as a strategy to facilitate RCW population recovery (USFWS, 2003), and individuals have been artificially moved throughout their range (Allen, Franzreb, & Escano, 1993; Carrie, Conner, Rudolph, & Carrie, 1999; Costa & DeLotelle, 2006; Cox & McCormick, 2016; Connor, Rodolph, & Bonner, 1995; Franzreb, 1999; Haig et al., 1993a; Rudolph, Conner, Carrie, & Schaefer, 1992). While more detailed analyses are still required, it remains feasible that the cumulative effects of translocations over the decades following their initiation may have reversed the loss of genetic diversity that was detected in this subset of the species' range.

Based on the mtDNA analysis, RCW have lost ~25%–30% of their haplotypes over the past century (*Ar*_mtDNA_ from “All Data” row in Table 1). This loss did not appear to include any phylogenetically distinct lineages (Figure 2) that might correspond to separate Evolutionarily Significant Units (Crandall, Bininda‐Emonds, Mace, & Wayne, 2000; Moritz, 1994). Instead, the temporal haplotype networks created for each time period had relatively similar star‐like topologies, and primary differences among the three networks could be attributed to the differences in sample sizes among time points (i.e., more haplotypes revealed in the 2010–2014 data due to a ~fivefold larger number of analyzed individuals).

Prior analyses from the 1990s highlighted significant genetic structure among RCW populations. Stangel et al. (1992) estimated an *F*_st_ of 0.14 in allozyme analyses of 26 populations while Haig, Rhymer, et al. (1994) estimated an *F*_st_ of 0.19 using RAPD markers from 14 populations. Haig et al. (1996) later revisited the previous RAPD analyses and included additional populations from Florida, resulting in an *F*_st_ of 0.21 that was comparable to that detected in the earlier investigation (Haig, Rhymer, et al., 1994). Our analyses of new microsatellite and mtDNA data based on samples from a similar time frame (samples from 1992–1995) produced similar results and illustrated significant genetic structure at multiple different spatial scales (Table 5). However, our use of data from more contemporary (2010–2014) and historical (pre‐1970) samples provided new insights that were not apparent from the single time point snapshots achieved in prior studies. Specifically, the mtDNA data show that changes in genetic structure have occurred in concert with the loss of genetic diversity that occurred prior to the 1990s (Table 5). Historically, RCW appears to have had continuous populations and panmictic genetic structure based on the negative point estimates of *F*_ST_ that were identified in the pre‐1970s mtDNA data. This widespread panmictic population has transitioned more recently into a discontinuously distributed species with isolated populations and reduced gene flow based on the low, but significant, population differentiation observed at later time points in both the mtDNA and microsatellite data. This result may indicate that RCW are reluctant to move among fragmented habitat patches. Furthermore, as with the analyses of genetic diversity, the 1992–1995 and 2010–2014 data suggest that there has been little overall change in genetic differentiation patterns since that time and may indicate a secondary benefit of management interventions such as translocations beyond the demographic impacts that have been observed in monitoring studies. A more thorough understanding of this outcome may be possible through future analyses based on the complete history of the RCW translocation program and documentation of source and recipient populations used for translocation purposes.

Our study is among a growing list of investigations that use historical DNA samples to identify genetic changes that have occurred in natural populations. These approaches are powerful, as they may provide insights not only about trends over time, but can also help determine if management actions are having the desired effects at the genetic level (Frankham et al., 2017; Haig et al., 2011; Schwartz et al., 2007). At this time, there is a need to better determine the effects of RCW management actions beyond the demographic impacts that have been previously documented. In future investigations, closer examination of over 20 years of translocation data, habitat information, and other demographic factors may provide a better understanding of recent genetic changes that have occurred in RCW. Such analyses may help identify secondary repercussions of management actions beyond the beneficial demographic impact that has been previously documented.

## CONFLICT OF INTERESTS

None declared.

## AUTHOR CONTRIBUTIONS

SMH and JRW conceptualized the project. SMH performed project oversight. JTV and TDM collected the data. JTV and MPM analysed the data. All authors were involved in interpretation of analysis results. All authors were involved in manuscript preparation and development of final content.

## Data Availability

DNA sequences: Genbank accessions MK253579–MK253645. Final Raw Data Sets: Data for this study are available as a U.S. Geological Survey data release (https://doi.org/10.5066/P9N6Z9W5).
