# Supplementary material for: Changes in genetic diversity and differentiation in Red‐cockaded woodpeckers (Dryobates borealis) over the past century
Source: Ecol Evol. 2019 Apr 8;9(9):5420–32. doi: 10.1002/ece3.5135 (PMC6509371; doi:10.1002/ece3.5135)
Supplement: Supplementary file 1 [file ECE3-9-5420-s001.docx]

Appendix S1. Sampling locations, geographic coordinates, regional grouping assignment, and ecoregion assignment for Red-cockaded woodpecker sampling periods. Regional grouping codes correspond to numbered regions illustrated in Fig. 1. Ecoregion codes are as follows: WGCP = West Gulf Coastal Plain; UWGCP = Upper West Gulf Coastal Plain; EGCP = East Gulf Coastal Plain; UEGCP = Upper East Gulf Coastal Plain; GCPAM = Gulf Coast Plains and Marshes; CUMB = Cumberlands; SAND = Sandhills; MACP = Mid Atlantic Coastal Plain; SACP = South Atlantic Coastal Plain; SCF = South Central Florida; GCPAM = Gulf Coast Prairies and Marshes. NF=National Forest, NWR = National Wildlife Refuge, SF = State Forest, WMA = Wildlife Management Area.

|  |  |  |  |  | Sample Size (n) | | | | |
| --- | --- | --- | --- | --- | --- | --- | --- | --- | --- |
|  | Regional |  |  |  | mtDNA | | | microsatellites | |
| Sample location | Group | Ecoregion | longitude | latitude | pre-1970 | 1992-1994 | 2010-2014 | 1992-1994 | 2010-2014 |
| Angelina NF | 1 | WGCP | -94.21 | 31.28 | 1 | 7 |  | 7 |  |
| Apalachicola NF/St Marks NWR/Tates Hell SF | 3 | EGCP | -84.63 | 30.17 | 1 | 6 | 19 | 4 | 19 |
| Avon Park Air Force Range | 3 | SCF | -81.26 | 27.72 | 4 | 7 | 4 | 7 | 4 |
| Babcock-Webb WMA | 3 | SCF | -81.83 | 26.87 |  | 7 |  | 7 |  |
| Bienville NF | 1 | UEGCP | -89.58 | 32.40 |  | 2 |  | 2 |  |
| Big Branch Marsh NWR | 1 | GCPAM | -89.92 | 30.28 | 3 |  |  |  |  |
| Big Cypress National Preserve | 3 | SCF | -81.07 | 26.00 |  | 5 | 16 | 5 | 9 |
| Blackwater River SF/Conecuh NF | - | EGCP | -86.80 | 30.92 |  | 5 | 17 |  | 17 |
| Camp Blanding | 3 | SACP | -81.98 | 30.03 |  |  | 12 |  | 13 |
| Marine Corps Base Camp Lejeune | 2 | MACP | -77.30 | 34.64 |  |  | 10 |  | 10 |
| Campbell Global Timberlands | 1 | WGCP | -93.17 | 30.65 |  |  | 4 |  | 4 |
| Carolina Sandhills NWR | 2 | SAND | -80.23 | 34.57 |  |  | 15 |  | 19 |
| Catahoula District/Winn District-Kisatchie NF | 1 | WGCP | -92.59 | 31.73 |  |  | 10 |  | 10 |
| Chickasawhay District-DeSoto NF | - | EGCP | -88.97 | 31.53 |  |  | 10 |  | 10 |
| Citrus Tract/Withlacoochee SF | 3 | SCF | -82.42 | 28.78 |  |  | 13 |  | 10 |
| Cook's Branch Conservancy | 1 | UWGCP | -95.67 | 30.28 |  |  | 5 |  | 5 |
| Corbett WMA | 3 | SCF | -80.32 | 26.87 |  | 5 | 5 | 5 | 5 |
| Croatan NF | 2 | MACP | -77.00 | 34.86 |  | 7 |  | 7 |  |
| Croom Tract/Withlacoochee SF | 3 | SCF | -82.28 | 28.59 | 1 |  | 9 |  | 9 |
| Crowell Lumber/Evangeline Unit-Kisatchie NF | 1 | WGCP | -92.57 | 31.22 |  |  | 29 |  | 29 |
| Daniel Boone NF | 2 | CUMB | -84.36 | 36.98 | 8 | 3 |  | 3 |  |
| D'Arbonne NWR | 1 | UWGCP | -92.20 | 32.64 | 3 |  | 2 |  | 2 |
| DuPuis WMA | 3 | SCF | -80.55 | 26.97 | 1 |  | 9 |  | 8 |
| Eglin Air Force Base | - | EGCP | -86.64 | 30.58 |  | 1 | 12 | 1 | 14 |
| Enon and Sehoy Plantations | - | EGCP | -85.49 | 32.19 |  |  | 9 |  | 9 |
| Felsenthal NWR and Huttig | 1 | UWGCP | -92.19 | 33.11 |  |  | 10 |  | 10 |
| Fort Benning | - | SAND | -84.82 | 32.39 |  |  | 13 |  | 13 |
| Fort Gordon | 2 | SAND | -82.22 | 33.36 |  |  | 5 |  | 5 |
| Fort Jackson | 2 | SAND | -80.83 | 34.06 |  |  | 13 |  | 13 |
| Fort Polk/Vernon Unit-Kisatchie NF | 1 | WGCP | -93.04 | 31.04 |  |  | 14 |  | 14 |
| Fort Stewart | - | SACP | -81.62 | 31.95 |  |  | 11 |  | 11 |
| Francis Marion NF/Bonneau Ferry WMA-Santee Coastal Reserve | 2 | MACP | -79.66 | 33.14 | 3 | 9 | 6 | 9 | 7 |
| Goethe SF | 3 | SCF | -82.61 | 29.19 |  |  | 14 |  | 13 |
| Homochitto NF | 1 | EGCP | -90.99 | 31.37 |  |  | 10 |  | 10 |
| Jackson-Bienville WMA/Weyerhaeuser | 1 | WGCP | -92.77 | 32.41 | 1 |  | 10 |  | 10 |
| Jones Ecological Research Center | - | EGCP | -84.47 | 31.22 |  |  | 10 |  | 10 |
| Manchester SF/Poinsett Range-Shaw Air Force Base | 2 | SAND | -80.49 | 33.80 |  |  | 10 |  | 10 |
| McCurtain County Wilderness Area | 1 | UWGCP | -94.68 | 34.32 | 2 |  | 7 |  | 7 |
| Military Ocean Terminal Sunny Point | 2 | MACP | -78.00 | 34.01 |  |  | 9 |  | 9 |
| Noxubee NWR | - | UEGCP | -88.84 | 33.25 |  | 4 | 8 | 4 | 8 |
| Oakmulgee District-Talladega NF | - | UEGCP | -87.40 | 32.95 |  |  | 10 |  | 10 |
| Ocala NF | 3 | SCF | -81.72 | 29.27 | 4 |  | 6 |  | 6 |
| Okefenokee NWR | 3 | SACP | -82.28 | 30.77 | 8 |  |  |  |  |
| Osceola NF | 3 | SACP | -82.48 | 30.25 | 2 | 2 | 11 | 2 | 11 |
| PalmettoPeartree Preserve | 2 | MACP | -76.08 | 35.95 |  |  | 10 |  | 10 |
| Peason Ridge/Kisatchie RD-Kistachie NF | 1 | WGCP | -93.20 | 31.42 |  | 9 | 18 | 9 | 17 |
| Picayune Strand SF | 1 | SCF | -81.61 | 26.11 |  |  | 8 |  | 8 |
| Piedmont NWR/Hitchiti Experimental Forest/Oconee NF | - | SAND | -83.71 | 33.11 |  |  |  |  |  |
| Piney Grove Preserve | 2 | MACP | -77.07 | 36.97 | 1 |  | 3 |  | 3 |
| Potlatch Corporation (Moro Big Pine) | 1 | UWGCP | -92.48 | 33.41 |  |  | 8 |  | 8 |
| Sabine NF | 1 | WGCP | -93.85 | 31.47 |  |  | 9 |  | 9 |
| Sam Houston NF | 1 | UWGCP | -95.58 | 30.52 | 7 | 5 |  | 5 |  |
| Sandhills East | 2 | SAND | -79.20 | 35.15 |  | 13 | 3 | 12 | 6 |
| Sandhills West | 2 | SAND | -79.57 | 35.02 |  | 7 | 3 | 7 | 1 |
| Savannah River Site | 2 | SACP | -81.56 | 33.29 |  | 11 | 7 | 11 | 7 |
| Shoal Creek RD-Talladega NF | - | UEGCP | -85.59 | 33.79 |  |  | 12 |  | 12 |
| Silver Lake WMA | - | EGCP | -84.72 | 30.80 |  |  | 9 |  | 10 |
| Three Lakes WMA/Triple N Ranch WMA/Bull Creek WMA | 3 | SCF | -81.04 | 28.00 |  | 8 | 9 | 8 | 9 |
| Warren Prairie | 1 | UWGCP | -91.96 | 33.56 |  |  | 3 |  | 4 |
| Weyerhauser North | 1 | WGCP | -93.57 | 33.00 |  |  | 9 |  | 9 |
| WG Jones SF | 1 | UWGCP | -95.49 | 30.22 |  |  | 3 |  | 3 |
| Total |  |  |  |  | 50 | 123 | 501 | 115 | 499 |
