# Supplementary material for: Changes in genetic diversity and differentiation in Red‐cockaded woodpeckers (Dryobates borealis) over the past century
Source: Ecol Evol. 2019 Apr 8;9(9):5420–32. doi: 10.1002/ece3.5135 (PMC6509371; doi:10.1002/ece3.5135)
Supplement: Supplementary file 2 [file ECE3-9-5420-s002.docx]

Appendix S2. Collection information associated with 50 Red-cockaded Woodpecker toepad samples obtained from museum collections. See Appendix I for Ecoregion abbreviation information. Institutional abbreviations are as follows. AMNH: American Museum of Natural History; CM: Carnegie Museum; MVZ: Museum of Vertebrate Zoology at Berkeley; LSUMZ: Louisiana State University Museum of Zoology; CUMV: Cornell University Museum of Vertebrates; UMMZ: University of Michigan Museum of Zoology; CAS-ORN: California Academy of Sciences Ornithology; TCWC/BRTC: Texas Cooperative Wildlife Collection/Texas A&M Biodiversity Research and Teaching Collections; OMNH: Sam Noble Oklahoma Museum of Natural History; FMNH: Field Museum of Natural History.

| Lab ID | State | Location | Regional Group | Ecoregion | Date | Inst. | Cat. No. |  |
| --- | --- | --- | --- | --- | --- | --- | --- | --- |
| MS01 | FL | Okefenokee_NWR | 3 | SACP | 11/27/1906 | AMNH | 363745 |  |
| MS02 | FL | Okefenokee_NWR | 3 | SACP | 12/10/1906 | AMNH | 363746 |  |
| MS03 | FL | Avon_Park Air Force Range | 3 | SCF | 2/19/1924 | CM | P162262 |  |
| MS04 | FL | Avon_Park | 3 | SCF | 2/19/1924 | CM | P162261 |  |
| MS05 | FL | Avon_Park | 3 | SCF | 4/2/1907 | AMNH | 363748 |  |
| MS15 | FL | Apalachicola/St_Marks/TatesHell | 3 | EGCP | 7/9/1941 | MVZ | 84038 |  |
| MS17 | FL | Ocala_NF | 3 | SCF | 12/30/1904 | AMNH | 363778 |  |
| MS18 | FL | DuPuis WMA | 3 | SCF | 1/30/1921 | AMNH | 755501 |  |
| MS24 | FL | Osceola_NF | 3 | SACP | 1/31/1942 | LSUMZ | 6471 |  |
| MS25 | FL | Osceola_NF | 3 | SACP | 2/2/1942 | LSUMZ | 6472 |  |
| MS27 | FL | Ocala_NF | 3 | SCF | 4/14/1905 | AMNH | 363768 |  |
| MS30 | FL | Ocala_NF | 3 | SCF | 2/2/1881 | AMNH | 363770 |  |
| MS32 | FL | Ocala_NF | 3 | SCF | 3/8/1881 | AMNH | 363772 |  |
| MS35 | FL | Avon_Park | 3 | SCF | 3/13/1889 | AMNH | 39022 |  |
| MS38 | FL | Croom_Tract/Withlacoochee_SF | 3 | SCF | 5/24/1912 | AMNH | 363774 |  |
| MS43 | GA | Okefenokee_NWR | 3 | SACP | 6/3/1912 | CUMV | 6629 |  |
| MS44 | GA | Okefenokee_NWR | 3 | SACP | 7/12/1922 | CUMV | 6628 |  |
| MS45 | GA | Okefenokee_NWR | 3 | SACP | 7/11/1922 | CUMV | 6627 |  |
| MS46 | GA | Okefenokee_NWR | 3 | SACP | 7/18/1921 | CUMV | 6630 |  |
| MS47 | GA | Okefenokee_NWR | 3 | SACP | 12/27/1898 | AMNH | 363781 |  |
| MS48 | GA | Okefenokee_NWR | 3 | SACP | 1/26/1893 | AMNH | 704380 |  |
| MS49 | KY | Daniel_Boone_NF | 2 | CUMB | 7/9/1948 | UMMZ | 211715 |  |
| MS51 | KY | Daniel_Boone_NF | 2 | CUMB | 7/7/1948 | UMMZ | 211707 |  |
| MS53 | KY | Daniel_Boone_NF | 2 | CUMB | 10/4/1951 | UMMZ | 211713 |  |
| MS55 | KY | Daniel_Boone_NF | 2 | CUMB | 2/5/1950 | UMMZ | 211710 |  |
| MS56 | KY | Daniel_Boone_NF | 2 | CUMB | 2/4/1950 | UMMZ | 211711 |  |
| MS57 | KY | Daniel_Boone_NF | 2 | CUMB | 4/29/1949 | UMMZ | 211712 |  |
| MS58 | KY | Daniel_Boone_NF | 2 | CUMB | 10/7/1951 | UMMZ | 211714 |  |
| MS59 | KY | Daniel_Boone_NF | 2 | CUMB | 11/21/1948 | UMMZ | 211717 |  |
| MS60 | LA | Jackson Parish | 1 | WGCP | 5/12/1949 | LSUMZ | 19497 |  |
| MS64 | LA | Big_Branch_Marsh | 1 | GCPAM | 2/22/1904 | CAS-ORN | 45207 |  |
| MS65 | LA | Big_Branch_Marsh | 1 | GCPAM | 2/5/1904 | CAS-ORN | 45209 |  |
| MS66 | LA | Big_Branch_Marsh | 1 | GCPAM | 2/22/1904 | CAS-ORN | 45210 |  |
| MS68 | LA | D'Arbonne_NWR | 1 | UWGCP | 8/30/1940 | LSUMZ | 4040 |  |
| MS70 | LA | D'Arbonne_NWR | 1 | UWGCP | 12/27/1940 | LSUMZ | 4589 |  |
| MS71 | LA | D'Arbonne_NWR | 1 | UWGCP | 12/23/1968 | TCWC/BRTC | 7722 |  |
| MS72 | OK | McCurtain_County_Wilderness_Area | 1 | UWGCP | 1/25/1969 | OMNH | 6491 |  |
| MS73 | OK | McCurtain_County_Wilderness_Area | 1 | UWGCP | 6/26/1925 | OMNH | 8886 |  |
| MS74 | SC | Francis_Marion/Bonneau_Ferry-Santee_Coastal_Reserve | 2 | MACP | 5/7/1907 | AMNH | 99482 |  |
| MS75 | SC | Francis_Marion/Bonneau_Ferry-Santee_Coastal_Reserve | 2 | MACP | 7/6/1912 | FMNH | 139828 |  |
| MS76 | SC | Francis_Marion/Bonneau_Ferry-Santee_Coastal_Reserve | 2 | MACP | 7/6/1912 | FMNH | 139829 |  |
| MS77 | TX | Angelina NF | 1 | WGCP | 6/21/1964 | MVZ | 153299 |  |
| MS78 | TX | Sam_Houston_NF | 1 | UWGCP | 1/10/1917 | AMNH | 363785 |  |
| MS79 | TX | Sam_Houston_NF | 1 | UWGCP | 1/11/1917 | AMNH | 363786 |  |
| MS80 | TX | Sam_Houston_NF | 1 | UWGCP | 1/17/1917 | AMNH | 363787 |  |
| MS81 | TX | Sam_Houston_NF | 1 | UWGCP | 1/10/1916 | AMNH | 363790 |  |
| MS82 | TX | Sam_Houston_NF | 1 | UWGCP | 9/6/1937 | TCWC/BRTC | 871 |  |
| MS86 | TX | Sam_Houston_NF | 1 | UWGCP | 8/11/1936 | TCWC/BRTC | 157 |  |
| MS87 | TX | Sam_Houston_NF | 1 | UWGCP | 7/10/1936 | TCWC/BRTC | 158 |  |
| MS88 | VA | Piney_Grove_Preserve | 2 | MACP | 12/30/1887 | AMNH | 96427 |  |
