# Supplementary material for: Changes in genetic diversity and differentiation in Red‐cockaded woodpeckers (Dryobates borealis) over the past century
Source: Ecol Evol. 2019 Apr 8;9(9):5420–32. doi: 10.1002/ece3.5135 (PMC6509371; doi:10.1002/ece3.5135)
Supplement: Supplementary file 3 [file ECE3-9-5420-s003.docx]

Appendix S3. Histogram of collection dates (years) for museum samples included in analyses. 95% of samples (45) were collected before 1950.
