# Supplementary material for: Changes in genetic diversity and differentiation in Red‐cockaded woodpeckers (Dryobates borealis) over the past century
Source: Ecol Evol. 2019 Apr 8;9(9):5420–32. doi: 10.1002/ece3.5135 (PMC6509371; doi:10.1002/ece3.5135)
Supplement: Supplementary file 4 [file ECE3-9-5420-s004.docx]

Appendix S4. Primer pairs used to amplify and sequence a fragment of the mitochondrial control region from Red-cockaded Woodpeckers.

| Amplicon | Primer |  | Amplicon | Annealing | Extension |
| --- | --- | --- | --- | --- | --- |
| Name | Name | 5’-3’ Sequence | Size (bp) | Temperature (°C) | Time |
| msCR1 | CB1f | TGCCAATCTCCTTATCCTCAC | 240 | 51 | 30 secs |
|  | new_CR1Er | AAGTACATAGGTTAGACATGAATG |  |  |  |
| msCR2 | CR1Df | TGTAAACCAAAGATTGAAGATACACA | 270 | 51 | 30 secs |
|  | new_550r | ACTGCCGTGTGGTAGGATAA |  |  |  |
| msCR3 | CR1Bf | CCGGTGTAAGTCACCATCCT | 240 | 51 | 30 secs |
|  | new_700r | TGGGCCTGAAGCTAGTAACG |  |  |  |
| msCR4 | new_650f | TCACCTCACGTGAAATCAGC | 130 | 51 | 30 secs |
|  | CR1Ar | CACGGACGAAAATGGTGATA |  |  |  |
| msCR2a | NEW_80f | TTCTCCTCCCTCTTGTCAGC | 100 | 53.5 | 30 secs |
|  | new_CR1Er | AAGTACATAGGTTAGACATGAATG |  |  |  |
| CBCR1a | CB1_f | TGCCAATCTCCTTATCCTCAC | 800 | 51.6 | 50 secs |
|  | CR1Ar | CACGGACGAAAATGGTGATA |  |  |  |
| CR8b | 10f2 | CTTTATCATTATCGGCCAACTG | 800 | 51.3 | 1 min |
|  | 680r | GTGCCAAAGGTTGGAATG |  |  |  |
