# Supplementary material for: Changes in genetic diversity and differentiation in Red‐cockaded woodpeckers (Dryobates borealis) over the past century
Source: Ecol Evol. 2019 Apr 8;9(9):5420–32. doi: 10.1002/ece3.5135 (PMC6509371; doi:10.1002/ece3.5135)
Supplement: Supplementary file 5 [file ECE3-9-5420-s005.docx]

Appendix S5.


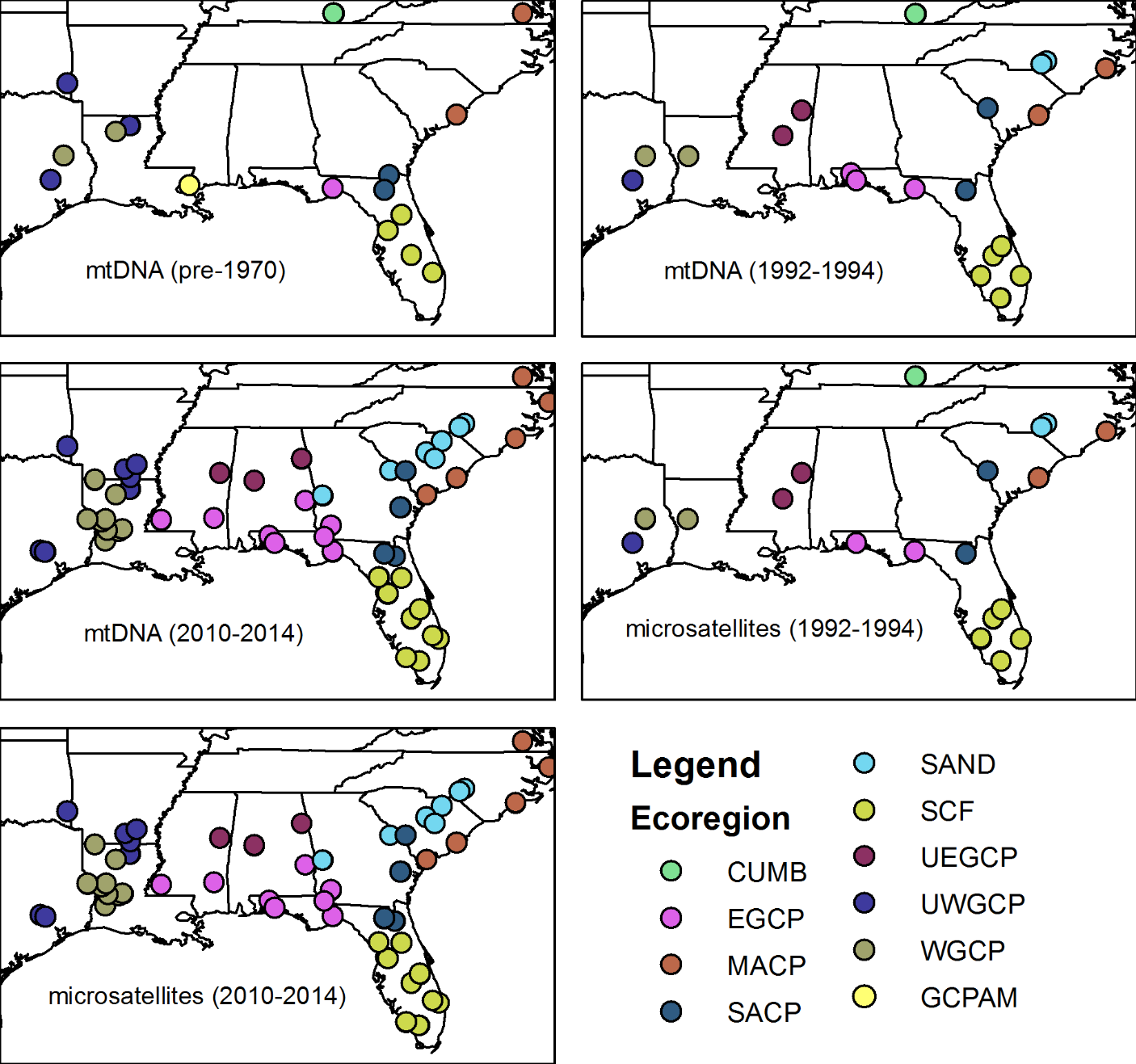


Map highlighting the spatial distribution of sample locations coded by Ecoregion. Legend codes are as follows: WGCP = West Gulf Coastal Plain; UWGCP = Upper West Gulf Coastal Plain; EGCP = East Gulf Coastal Plain; UEGCP = Upper East Gulf Coastal Plain; CUMB = Cumberlands; SAND = Sandhills; MACP = Mid Atlantic Coastal Plain; SACP = South Atlantic Coastal Plain; SCF = South Central Florida; GCPAM = Gulf Coast Plains and Marshes.
