# Supplementary material for: Changes in genetic diversity and differentiation in Red‐cockaded woodpeckers (Dryobates borealis) over the past century
Source: Ecol Evol. 2019 Apr 8;9(9):5420–32. doi: 10.1002/ece3.5135 (PMC6509371; doi:10.1002/ece3.5135)
Supplement: Supplementary file 6 [file ECE3-9-5420-s006.docx]

Appendix S6. Results of analyses using the program STRUCTURE (Pritchard et al. 2000) to analyze the 1992-1995 and 2010-2014 microsatellite data. Results of analyses indicate the presence of K = 1 genetic clusters because the likelihood score of the K = 1 model was largest.

|  | Mean LnP(K) | |
| --- | --- | --- |
| K | 1992-1995 | 2010-2014 |
| 1 | -1569.28 | -6435.78 |
| 2 | -1609.78 | -6641.54 |
| 3 | -1624.48 | -6904.84 |
| 4 | -1604.68 | -7158.16 |
| 5 | -1615.94 | -7452.94 |
